# Psychometric properties of the end-of-life care decision inventory (EOL-CDI): a mixed-methods study

**DOI:** 10.1186/s12955-022-01952-8

**Published:** 2022-03-24

**Authors:** Shinmi Kim, Insook Lee, Sun-Woo Hong, Su-Jin Koh

**Affiliations:** 1 Department of Nursing, Changwon National University, C.P.O. Box 51140, Changwon, Korea; 2 Department of Emergency Medical Services, Daejeon University, Daejeon, Korea; 3 Division of Haematology and Oncology, Department of Internal Medicine, Ulsan University Hospital, Ulsan University College of Medicine, Ulsan, Korea

**Keywords:** Terminal care, Decision-making, Cognitive interview, Psychometric tests

## Abstract

**Background:**

End-of-life care decision-making has become important to support dignity and quality of life for patients who are facing death in Korea, along with the enactment of the Life-Sustaining Treatment Act in 2018. However, it seems that the concepts and policies related to the law are not yet familiar to health care providers or the general public. This unfamiliarity can hinder efficient end-of-life care discussions. Therefore, the purpose of this study was to propose a valid and reliable tool to explore the level of understanding of concepts and attributes related to end-of-life care decisions.

**Methods:**

This is a mixed-methods study design. A relevant law and literature analysis, expert consultation, cognitive interviews of 10 adults, and cross-sectional survey for psychometric tests using data from 238 clinical nurses were performed to update a tool developed before the life-sustaining treatment Act was enacted in Korea.

**Results:**

29 items of the draft version were polished in terms of literacy, total length, and scoring method via cognitive interviews and finalized into 21 items through psychometric tests and expert consultations. The 21 items conformed to the Rasch unidimensional paramenters.

**Conclusion:**

A tool to identify the level of understanding of concepts related to end-of-life care decisions was proposed through a rather rigorous process to ensure feasibility and validity/reliability. We recommend the proposed tool to apply to the adult population and nurses for evaluation and educational purposes.

## Background

In Korea, as of February 2018, the “Hospice, Palliative Care, and Life-sustaining Treatment Act for Patients in the End of Life” (LST Act) was enforced. This law aims to support the dignity and values of human beings at the end of their lives and stipulate the contents related to hospice palliative care and LST. While it is well recognized that care for the well-being should be continued for patients in the EOL period, aggressive and invasive care in the EOL period has long been a subject of debate [1]. LST refers to a medical procedure performed to maintain the patient's life without a therapeutic effect [2]. And issues related to withholding/withdrawal of LST have been controversial legally and ethically [1]. In this debate, a patient’s autonomy and self-determination are constantly acknowledged [3, 4] and the LST Act in Korea also respect their autonomy and self-determination.

To ensure self-determination in EOL care decision-making process, advance care planning (ACP) is brought up because it helps improve the quality of life during the EOL period [5]. EOL discussions for ACP are processes in which healthcare providers, patients, and their family members communicate and make decisions in advance along with the patient's value and preference as well as the patient's disease state [6].

For efficient ACP, shared decision-making is recommended, which is believed to make mutual understanding possible among stakeholders [7]. And upon mutual understanding, EOL care decision that is in the best interests of the patient can be determined and documented. Such documents usually include advance directives (AD) and physician’s order of life-sustaining treatment (POLST), and the LST Act in Korea also presents these two documents, so understanding of these is also required in relation to EOL care decisions.

However, the concepts and policies related to the LST Act are unfamiliar not only to the general public but also to healthcare providers. And there is a gap between theoretical concept and actual practice in terms of definitions and properties. Therefore, it is necessary to understand the basic yet pracatical contents required in clinical settings.

There has been a continuing interest in the level of understanding of EOL care decisions for various groups in Korea, and thus, the authors presented a tool for adults in a previous study [8]. Since then, this specific tool has been steadily used for diverse respondent groups by various researchers in Korea. However, the situation in Korea has changed significantly and this old version is no longer useful. In addition, it is difficult to find a tool that reflect the current situation in Korea. Therefore, revision of this old tool became an urgent task.

In particular, as the law and policies began to be implemented in clinical settings, practical information became more important than theoretical knowledge. Especially, ‘basic’ information or attributes of EOL care circumstances, including ACP documents of AD/POLST and EOL care options, are keys for efficient EOL discussion. We emphasize 'basic' because it is a kind of maginot line information necessary for EOL discussions among stakeholders regardless of education level, age, etc. Above all, EOL care decision-making has become a part of people’s life with the LST Act and identifying the level of understanding about EOL care decisions using a valid and practical tool is called for.

In Korea, the profession that performs the most any related tasks in the process of applying the law in clinical practice is known to be a nurse [9]. Besides, in the process of nurses’ interaction with patients and their family members in relation to EOL care decisions, whether formally or informally, nurses can influence decision-making itself or attitude toward EOL care [10, 11], so nurses’ level of knowledge about EOL care decisions also is important. Therefore, this study attempted to update an existing tool by targeting, but not limited to, the general public and clinical nurses.

The purpose of this study was to present a tool to identify the level of understanding about contents directly related to EOL care decision-making in clinical settings such as terminal/EOL state, ACP documents of AD/POLST and EOL care options. Specifically, the first objective of this study was to propose a tool with adequate literacy and applicability, and the second objective was to secure validity and reliability of this tool. Finally, we hypothesized that an updated new version would have stronger validity, and applicability for actual research and practice with items that include essential and practical contents.

## Methods

### Study design

We adopted qualitative and quantitative approaches with a mixed-method design for research purposes. A cognitive interviews were performed to assess literacy and feasibility, and a crossectional survey was performed to examine reliability and validity.

This study is one part of a larger project to identify the understanding level of information related to EOL care decisions among various populations in Korea, including medical personnel and the general public. In order to make this project meaningful, as the first step of the entire project, a study was conducted to present a valid and reliable tool through the revising process of the old version [8]. Therefore, validity and reliability of this revised tool can be further confirmed upon completion of the entire project, including adult and elderly population.

### Study procedures

**Fig. 1:**
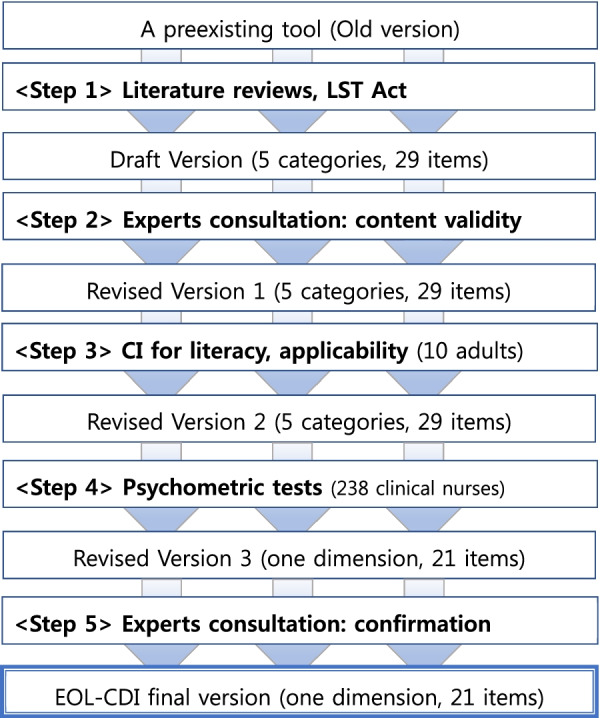
Five steps of the study

**Fig. 2:**
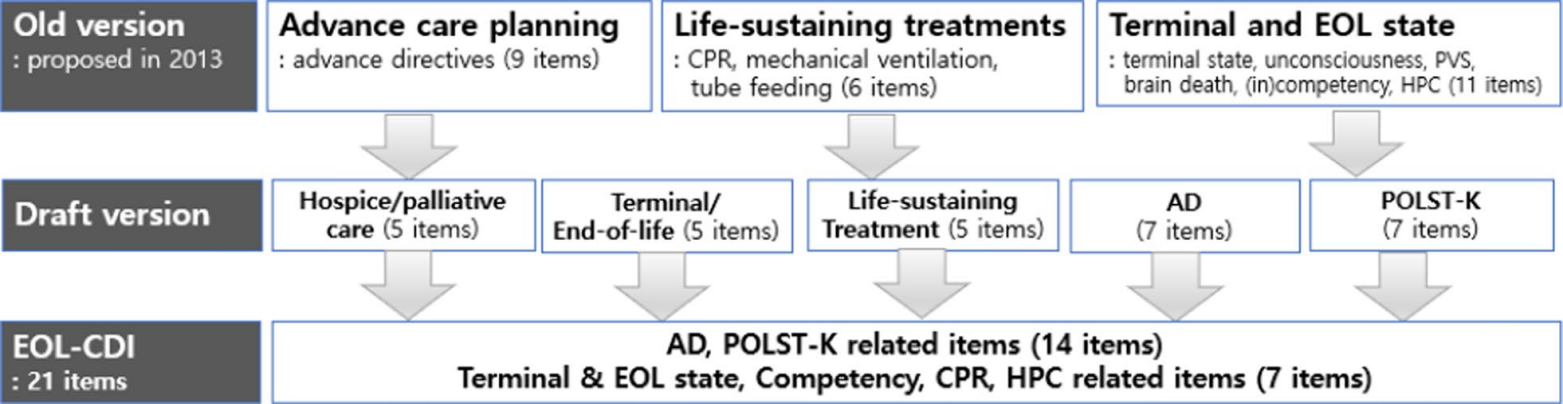
The flow of conceptual attribution of the multiple standardized phases of the study. *AD* advance directives, *CPR* cardiopulmonary resuscitation, *EOL-CDI* end-of-life care decision inventory, *EoL* end-of-life, *HPC* hospice/palliative care, *POLST-K* physician’s order of life-sustaining treatment Korean, *PVS* persistent vegetative state

This study was carried out in five steps; (1) modifying an old tool into the [Draft Version] based on LST Act of Korea, clinical situations and relevant literatures, (2) performing content validity evaluation and correction through multiple expert panel consultations via face to face and/or on-line meetings [Revised Version 1], (3) polishing items and a tool itself through cognitive interview results [Revised Version 2], (4) rearranging or excluding items using quantitative data analysis [Revised Version 3], (5) confirming [Final version] via consensus process of authors and expert panels (Figs. 1, 2).

For advice on the draft, revised versions and entire revision processes, a 15-expert panel was organized with five oncologists, five hospice palliative care experts, and five nurses. The reason for inviting experts currently working in clinical settings in the panel was that practical advice was critical.

#### Preparing draft version (step 1)

To prepare a draft, items like persistent vegetative state were omitted since they are neither in LST Act nor applicable in practice by the law, and POLST which was totally new policy in Korea was added. Items related to artificial feeding were also excluded because they were not negotiable in Korean society and had to be provided. As a result, a total of 29 items under five categories of hospice palliative care, terminal/EOL, LST, AD, POLST was proposed for the further revision process (Fig. 2). All items were prepared in consideration of the readability and clarity, and three answering option of "yes", "no", “don't know” was offered upon expert panel’s opinion. Experts had suggested adding a "don't know" option as some people can answer by guessing or unmarking "yes" or "no" if they didn’t know the answer, both of which could lead to inaccurate results. And, of course, the case of selecting “don’t know” was treated as a not-correct answer.

#### Validity consultations (step 2)

A draft version was sent to all expert panel members for content validity consultations. Meetings for comments on the draft were conducted face-to-face and/or online as needed. Categories, items, and draft itself were modified upon panel members’ opinions (Revised Version 1).

#### Cognitive interview (CI, step 3)

In order to ensure the appropriateness of response time, literacy, expression, and overall length, arrangement of items, CI was conducted. Usually, the subject of CI should be a person with the same characteristics as the subject of the actual survey; therefore, adults from diverse age groups were recruited. Inclusion criteria for CI participants were voluntarism and communication capability. Potential participants were excluded if they had serious physical problems, cognitive and/or mental dysfunction. While the number of subjects for CI is not specified, 5–10 people are usually acceptable [12]. Therefore, in this study, 10 people participated through a convenient sampling of 1–3 men and women by age group in their 20 s through 70 s from the local areas.

Two researchers (SK, IL) operated all the interviews together, one (SK) led the interview, and the other (IL) helped and/or co-hosted the session recording on-site notes and asking supplementary questions. All subjects were interviewed at their convenience and more than one interview was carried out when it was necessary.

Prior to CI, three researchers (SK, IL, SWH) prepared the interview protocol together, and two researchers who actually run the interview had trained themselves to familiarize ‘Revised Version 1’ and CI protocol. CI protocol included following elements in order: general guide for interviewers; a priori and cognitive interview preparation including guidance on behavior upon arrival of subject and instructions to be read to the subject; interview procedure (introduction including ‘think-aloud’ practice, presenting a EOL-CDI ‘Revised Version 1’, cognitive interview tips using ‘think-aloud’ and ‘probing’, and wrap up).

One researcher who was conducting the interview applied the ‘think aloud’ and ‘probing’ methods as appropriate [13], and the details are as follows. First, a leading interviewer requested an interviewee to go through ‘Revised Version 1’ and then asked questions like “How do you think about it?”, “Do you have any questions about it?”, “Could you please explain what item # 1 means?”, and so on. Subsequently, the interviewer asked the subject about their thoughts and opinions on the ‘Revision Version 1’ as a whole as well as the items and terms presented in it. Any difficult or incomprehensible parts in terms or expressions also were solicited. Each CI session took about 50 to 90 min, and after each interview, the researchers conducted a debriefing. Afterwards item arrangement, words, expressions, and version itself were polished based on analysis (Revised Version 2).

#### Quantitative tests (step 4)

Psychometric tests included construct validity and internal consistency test, and difficulty and discrimination of the items also were examined. Since this part of study was cross-sectional design, the relevant aspects of this part of study were informed by the STROBE checklist. The subjects for these procedures were clinical nurses. Nurse participants were recruited from diverse units of six hospitals located in three different areas of Korea. Nurse respondents were recruited without being limited to units where EOL care is more common, such as the oncology department. This is because the purpose of this study was to identify the understanding level of general clinical nurses’s regardless of their familiarity with the EOL care decision issues. Therefore, no specific condition for participation was applied except voluntarism.

The sample size for factor analysis requires a sample of 5–10 people per item number [14, 15], and since there are 29 items in the ‘Revised Version 2’, 150–300 people were required. On the other hand, in order to use the 2-parameter logistic model for item analysis, around 250 subjects were needed [16]. Therefore, in this study, 250 potential nurse participants were recruited considering all aspects. In addition to psychometric tests, the Rasch unidimensional measurement model [17] was applied since it is recommended to complement traditional psychometric approaches if a scale with invariant measurement properties supposed to be proposed [18].

#### Confirming final version (step 5)

After the quantitative analysis was completed, the results and the tentative final version were sent to the expert panel in advance and a meeting was held afterwards. For this meeting, researchers visited or invited expert panel members to discuss together, and experts who were unable to participate sent their opinions via email. The final version based on the experts’ opinions was open to all expert members and all authors for confirmation.

### Data collection

All qualitative and quantitative data were collected after approval of the project by the Institutional Review Board (IRB) of C University (No. 1040271-201905-HR-020). From September 2019 to January 2020, expert consultation, CI, quantitative data collection were performed in order.

### Data analysis

#### Cognitive interview

Three researchers separately reviewed all data, namely, field notes, debriefing data, and transcripts, to analyse CI data. Then, two researchers who had run the interviews modified ‘Revised version 1’ together based on analysis results. If there were any disagreements between them, the third researcher joined to reach an agreement. Upon agreement among the three researchers, ‘Revised Version 2’ was settled.

#### Quantitative data

Psychometric test analyses were performed using IBM SPSS Statistics 27.0 (IBM SPSS, Chicago, IL, USA), Exel, and Winstep 4.8.1 program (Winstep Inc., Chicago IL, USA). The score of each item was converted into a binary type (correct or incorrect), and the case of answering 'don't know' was considered as incorrect.

To test the difficulty and discrimination of the item, two methods were applied. First, SPSS and Excel were used to obtain the difficulty and discrimination of the item according to the classical theory. The item difficulty index according to the classical test theory is calculated using the number of correct answers as the numerator and the total number of subjects as the denominator (formula 1). Range of item difficulty index (P) is 0 ≤ P ≤ 1. And the item discrimination index according to the classical test theory is calculated using the difference between the number of correct answers in the upper performance group and the number of correct answers in the lower performance group (formula 2). Range of item discrimination index (R) is − 1 ≤ R ≤ 1. And then the KoreaPlus package of SPSS 27.0 and Winstep 4.6.1 were used to obtain the difficulty and discrimination of the item based on the item response theory (IRT).

1 $${\text{P}} = \frac{R}{N}$$

P: item difficulty, R: number of subjects who responsed right answer, N: total number of subjects

2 $$R = \frac{{P_{H} }}{{N_{H} }} - \frac{{P_{L} }}{{N_{L} }}$$

R: item discrimination, P_H_: number of subjects who responsed correct answer in high performance group, P_L_ number of subjects who responsed correct answer in low performance group, N_H_: total number of high performance group, N_L_: number of low performance group.

The validity of the tool was verified through the analysis of the fit of the model and the item fit of the Rasch model. A principal component analysis of residuals was performed to confirm the one-dimensionality assumption of the Rasch model, and the Rasch model fit of each item was confirmed using the mean square residual (MnSq) of the infit and outfit per each item, and point-measure correlation was calculated as well. The fit of the Rasch model can be judged to be appropriate if the statistical value is located between 0.5 and 1.5 centering on the expected value of 1.0 [19]. However, in this study, the standard is raised so that the infit MnSq and outfit MnSq values are 1.3 or more or less than 0.6 and the Z-value is less than − 2 or more than + 2.0, and items outside the criteria were judged as an inappropriate item and deleted since we want to develop good items that can be measured not only for medical personnel but also for general adults. The one-dimensionality assumption of the Rasch model is satisfied when the variance is at least 40%, and the first eigenvalue variance is less than 3.0 [20]. If the number of individual items per factor is too large, the measurement error increases, or false correlations between parameters tend to occur, and the model fit is deteriorated, which may impair the stability of the estimation. In general, three to four items per factor are recommended [21]. Based on these contents, the researchers organized the items, and the researchers reviewed the appropriateness of the items to be deleted based on the results of statistical analysis.

1. Inclusion of items

Decisions on the assignment of items to a factor were based on the following criteria:

- Item difficult by CTT < 80% or item difficult by IRT >  − 2.0 AND Item discrimination by CTT > 0.2 or Item discrimination by IRT > 0.0
- MnSq > 1.3 or < 0.6 AND Z-value > 2.0 or <  − 2.0 [19]
- Point-measure correlation > 0.3
- Rasch model’s unidemisionality: variance ≥ 40% and first eigenvalue variance < 3.0 [20]
- Three to four items per one factor [21]

Items met all criteria were included and differences between known groups confirmed for known-groups validity using t-test. Internal consistency was determined through person separation reliability to test the sensitivity of distinguishing high or low knowledge groups and person separation index to examine the degree to which study respondents could be differentiated into certain knowledge groups.

## Results

### Cognitive interview

A total of ten people participated in CIs (Table 1). Overall length, number of items, and answer options of ‘Revision Version 1’ were reported to be adequate in general. In the meantime, study participants tended to respond to the question items as if they were taking a test, stating as follows: *“It’s hard to answer because I don't know what this is……”* or *“I can't answer because I don't know if it is right or not……”* However, if a participant does not know the correct answer, it is also meaningful, so a strategy was required to allow respondents to respond freely. For that reason, the phrase was added to the description as follows: *“Please don't think long about each item and answer as it comes to your mind. If you don't know, you can mark ‘don’t know’.”* The overall arrangement of categories was changed in the order of hospice palliative care, terminal/EOL, LST, AD, and POLST, along with restatement in some words based on the subjects’ opinions.

**Table 1:** Characteristics of the participants for cognitive interviews (*N* = 10)

Participants	Gender	Age	Education level	Occupation
Participant 1	Male	25	High School	College student
Participant 2	Female	22	High School	College student
Participant 3	Female	22	High School	Service worker
Participant 4	Male	38	Master’s degree	Office worker
Participant 5	Female	42	Bachelor’s degree	Office worker
Participant 6	Male	46	Bachelor’s degree	Businessman
Participant 7	Male	47	Ph.D	Lecturer
Participant 8	Female	51	Bachelor’s degree	Public officer
Participant 9	Female	66	Middle school	None
Participant 10	Female	72	Elementary school	None
Mean ± SD		43.10 ± 17.29		

*Ph.D* doctor of philosophy, *SD* standard deviation

Examples of specific modifications are as follows. First, some expressions were changed in the hospice palliative care category due to misunderstanding (e.g., provision of LST → implementation of LST). Regarding the question that includes 'decision-making capacity', participants in their 60 s and 70 s were especially confused 'decision-making' with ‘doctor’s decision’, since Korean pronunciation of ‘decision-making’ and ‘doctor’ decision’ is the same. Therefore, Chinese characters (의사[意思]결정) were written as well. Regarding LST, concise expression was preferred, such as “This is a treatment that extends the duration of the patient's death process only.” without any additional explanation. AD-related items were revised according to the legal provisions.

Regarding POLST, participants complained about the item “If you complete POLST, all medical treatment will be stopped”, stating that “all medical care” was ambiguous. Instead, they sugggested “all medical care including pain relievers and antibiotics will be stopped.”, and this suggestion was retained.

### Psychometric tests

#### Study population

Data from 238 study participants were analyzed, excluding those who did not complete or did not respond out of 250 potential respondents. On average, study participants were 28.0 years old (± 5.3) and had worked in nursing for 62.8 months (± 63.43). In total, 47.4% worked in the medical-surgical unit, 31.1% in the intensive care unit or emergency room, 9.7% in oncology, and 11.8% in other departments. The proportions of participants with experience in the EOL care, LST Act education, and LST Act practice were 67.1%, 37.8%, and 64.6%, respectively (Table 2).

**Table 2:** General characteristics of the subjects for psychometric tests (*N* = 238)^*^

Variables	Categories	n (%)
Gender	Male	15 (6.3)
	Female	223 (93.7)
Age(yr)	< 30	186 (78.2)
	30–39	35 (14.7)
	≥ 40	17 (7.1)
Spouses	No	191 (80.6)
	Yes	46 (19.4)
Religious	No	153 (64.3)
	Yes	85 (35.7)
Education	Associate degree	19 (8.0)
	Bachelor’s degree	196 (82.4)
	Master’s degree and higher	23 (9.7)
Affiliation	General hospital	238 (100.0)
Wards	Internal medicine	47 (19.7)
	Surgical ward	66 (27.7)
	ICU	50 (21.0)
	ER	24 (10.1)
	Oncology	23 (9.7)
	Others(OBGY, OT, UR)	28 (11.8)
Position	Staff nurse	207 (87.0)
	Physician assistant	8 (3.4)
	Charge nurse	13 (5.5)
	Unit manager	10 (4.2)
Working experience (months)	< 12	25 (10.8)
	12–35	81 (34.9)
	36–59	52 (22.4)
	60–119	45 (19.4)
	≥ 120	29 (12.5)
EOL care experience	No	78 (32.9)
	Yes	159 (67.1)
LST Act education	No	148 (62.2)
	Yes	90 (37.8)
LST Act practice	No	84 (35.4)
	Yes	153 (64.6)
Involvement level in LST Act practice	Low	48 (31.6)
	Moderate	86 (56.6)
	High	18 (11.8)

*Missing data excluded

*SD* standard deviation, *ICU* intensive care unit, *ER* emergent room, *OBGY* obstetric and gynecology, *OT* ophthalmology, *URO* urology, *EOL* end-of-life, *LST* life-sustaining treatment

#### Item analysis

The results of item analysis on 29 items of ‘Revised Version 2’, including difficulty and discrimination tests of the items and the range of the item-total correlation (ITC), are shown in Table 3. The difficulty level by classical theory is as follows: 7 items (24.1%) of low difficulty (80–100% correct answer rate), 20 items (69.0%) of intermediate difficulty (20–80% correct answer rate), and 2 items (6.9%) of high difficulty (0–19% correct answer rate). The difficulty of the items through the item response theory is as follows: 12 very easy items less than − 2.0 (41.4%), 6 easy items less than − 2.0 to − 0.5 (20.7%), 6 intermediate items less than − 0.5 to 0.5 (20.7%), 2 difficult items less than 0.5–2.0 (6.9%), and 3 very difficult items above 2.0 (10.3%).

**Table 3:** Initial item analysis of EOL-CDI (29 items) (*N* = 238)

Items	Mean ± SD	Item difficulty index		Discrimination index	
		CTT	IRT	CTT	IRT
EOL-CDI item 1	0.97 ± 0.18	0.97	− 3.17	0.10	1.35
EOL-CDI item 2	0.90 ± 0.30	0.90	− 2.81	0.24	0.91
EOL-CDI item 3	0.94 ± 0.24	0.94	− 2.52	0.16	1.48
EOL-CDI item 4	0.47 ± 0.50	0.48	0.98	0.18	0.10
EOL-CDI item 5	0.69 ± 0.46	0.69	− 2.81	0.26	0.29
EOL-CDI item 6	0.63 ± 0.48	0.63	− 3.77	0.23	0.15
EOL-CDI item 7	0.76 ± 0.43	0.76	− 4.70	0.25	0.25
EOL-CDI item 8	0.32 ± 0.47	0.32	− 6.30	0.06	-0.12
EOL-CDI item 9	0.86 ± 0.35	0.86	− 2.76	0.23	0.71
EOL-CDI item 10	0.76 ± 0.43	0.77	− 2.35	0.26	0.53
EOL-CDI item 11	0.39 ± 0.49	0.39	2.14	0.27	0.22
EOL-CDI item 12	0.87 ± 0.33	0.87	− 2.72	0.23	0.79
EOL-CDI item 13	0.96 ± 0.20	0.96	− 2.52	0.14	1.87
EOL-CDI item 14	0.52 ± 0.50	0.52	− 0.14	0.42	0.47
EOL-CDI item 15	0.71 ± 0.45	0.71	− 3.12	0.28	0.30
EOL-CDI item 16	0.63 ± 0.48	0.63	− 0.39	0.67	1.78
EOL-CDI item 17	0.82 ± 0.39	0.82	− 1.07	0.57	2.59
EOL-CDI item 18	0.50 ± 0.50	0.50	0.06	0.54	1.04
EOL-CDI item 19	0.11 ± 0.31	0.11	3.50	0.24	0.66
EOL-CDI item 20	0.45 ± 0.50	0.45	0.26	0.66	1.25
EOL-CDI item 21	0.17 ± 0.38	0.17	2.80	0.25	0.60
EOL-CDI item 22	0.74 ± 0.44	0.74	− 0.69	0.74	2.59
EOL-CDI item 23	0.73 ± 0.45	0.73	− 0.81	0.51	1.66
EOL-CDI item 24	0.71 ± 0.45	0.71	− 0.82	0.61	1.40
EOL-CDI item 25	0.79 ± 0.41	0.79	− 1.04	0.60	2.03
EOL-CDI item 26	0.53 ± 0.50	0.53	− 0.10	0.56	1.05
EOL-CDI item 27	0.41 ± 0.49	0.41	0.47	0.61	0.97
EOL-CDI item 28	0.29 ± 0.46	0.29	0.90	0.57	1.31
EOL-CDI item 29	0.73 ± 0.45	0.73	− 0.78	0.66	1.77
Mean		0.63	− 1.18	0.38	1.03
SD			2.20		0.73

*SD* standard deviation, *EOL-CDI* end-of-life care decision inventory, *ITC* item-total correlation, *CTT* classic test theory, *IRT* item response theory

The discrimination degree of items tested by the classical theory was 5 items (17.2%) of low discrimination (less than 0.2), 11 items (38.0%) of intermediate discrimination (less than 0.2–0.4), and 13 items (44.8%) of high discrimination (more than 0.4). The degree of discrimination of the items through the IRT is as follows: 7 items (24.1%) with little discrimination power (less than 0.4), 3 items (10.3%) with low power (less than 0.4–0.7), 9 items (31.0%) with adequate power (less than 0.7–1.4), 4 items (13.8%) with high power (1.4 to less than 1.7), and 6 items (20.7%) with very high power (1.7 or more).

The Rash model based on the IRT was used to analyse the fitness of the item using 28 questions, excluding item 8 (questions related to the unconscious state) with negative discrimination, considering the degree of difficulty and discrimination using IRT.

#### Rasch unidimensional measurement model analysis

As a result of Rasch model analysis on 28 items were primarily organized (Table 4). The range of the point-measurement correlation coefficient for each item was 0.2–0.7, and the items outside the standard value of item fit (0.6–1.3) were item number 4 (If you receive HPC, LST won’t be provided), 5 (HPC is also available at home), 6 (Terminal disease can be cured with adequate treatment), 13 (A patient has right to decide LST). After excluding above four items, a Rasch model analysis of 24 items was conducted again. Consequently, item number 7 (Cancer is a terminal state), 11 (LST is a treatment that extends the duration of dying of patients who cannot recover), and 15 (If you are on a ventilator, a tube will be connected to the airway and breathe through a machine) were identified as inadequate and removed, and finally, appropriate items were identified as 21 items.

**Fig. 3:**
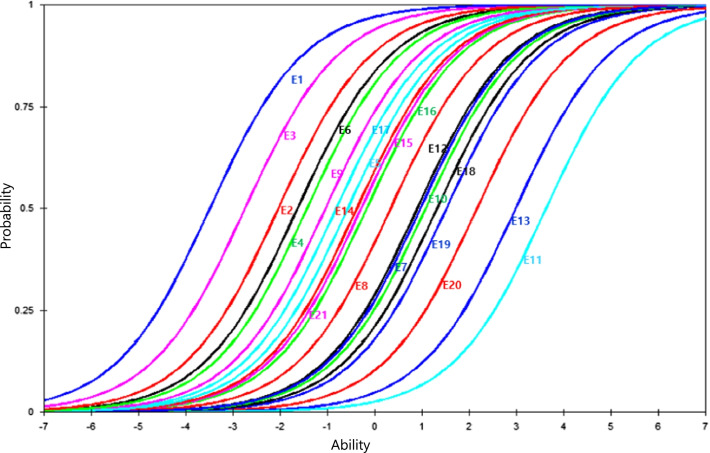
Item characteristics curve for final 21 items

1. *Goodness-of-fit of the model*
  Through principal component analysis of a 21-item version using the standardized residuals of the Rasch model, it was checked whether the one-dimensionality, the basic assumption of the Rasch model analysis, was satisfied. Through this, the validity of the inner structure of the scale can be confirmed. Analysis results with 21 items are listed in Table 5. Specifically, the variance explained by the Rasch measurement was 44.2%, and the eigenvalue of the first residual variance excluding the Rasch factor was 2.0, indicating that it supports one-dimensionality.
  The assumption of local independence was tested by checking residual correlations between items. In the tool revised in this study, all residual correlation coefficients were found to be less than 0.3, and item independence was satisfied. To confirmed the assumption of monotonicity, it was identified that the threshold values in the correct order appeared in all items through the person-item map.
2. *Goodness-of-fit of the items*
  The Rasch model was re-analyzed using 21 items to obtain the item's difficulty, item fit, separation reliability and separation index (Table 6). As a result, the range of the point-measurement correlation coefficient for each item was 0.2–0.7, and there were no items showing negative values. The items that exceeded the standard value of the item fit (0.6–1.3) of the infit MnSq value and the outfit MnSq value were one item (A patient in EOL period does not have decision-making capacity). The infit MnSq 1.3, Z-value 2.8, outfit MnSq 1.5, and Z-value 2.3 exceeded the standard value of the item fit (0.6–1.3), but the point-measure correlation was 0.3. Since it exceeded 0.3, it was decided not to remove it from the inventory. In addition, the item characteristic curve (ICC) was used to confirm the suitability of the item (Fig. 3). Figure 3 demonstrates the relationship between EOL-CDI and the probability of a correct response monotonically increases for the 21 EOL-CDI items. This means that the more awareness/knowledge a respondent has, the more likely they are to respond correctly to an item. Looking at the relative difficulty according to the position of the graph, item 11 is the most difficult item because it is the rightmost item on the y-axis, and item 1 is the easiest item because it is the leftmost item.
3. *Construct validity*
  The known-group validity was implemented to identify the differences between relevant groups. As a result, it was found that the group who had educated about LST Act (t = 4.6, *p* < 0.001), and the group with EOL care experience (t = 2.3, *p* = 0.024), and the group working in the setting practicing the LST Act (t = 3.1, *p* = 0.002) showed a significantly higher level of knowledge than the opposite groups, respectively. Thereby, the construct validity through the known-group validity was confirmed (Table 7).
4. *Reliability*

**Table 4:** Item fit statistics and point measure correlation of EOL-CDI (28 items)

EOL-CDI item	Logit	SE	Infit		Outfit		Point measure correlation	
			MnSq	Z	MnSq	Z	Observed	Expected
EOL-CDI item 1	− 3.17	0.40	0.90	− 0.20	0.70	− 0.36	0.42	0.34
EOL-CDI item 2	− 1.79	0.24	0.99	0.02	1.03	0.20	0.40	0.40
EOL-CDI item 3	− 2.45	0.30	0.79	− 0.88	0.97	0.06	0.49	0.37
EOL-CDI item 4	1.08	0.14	1.23	4.72	1.31	3.46	0.17	0.38
EOL-CDI item 5	− 0.01	0.16	1.22	2.72	1.30	2.73	0.24	0.43
EOL-CDI item 6	0.29	0.15	1.27	3.82	1.31	3.27	0.20	0.42
EOL-CDI item 7	− 0.45	0.17	1.23	2.23	1.27	1.91	0.25	0.43
EOL-CDI item 9	− 1.24	0.21	1.05	0.37	1.15	0.73	0.37	0.42
EOL-CDI item 10	− 0.48	0.17	1.15	1.50	1.19	1.83	0.23	0.35
EOL-CDI item 11	1.50	0.14	1.13	2.54	1.19	1.83	0.23	0.35
EOL-CDI item 12	− 1.42	0.22	1.10	0.72	1.08	0.41	0.34	0.41
EOL-CDI item 13	− 2.88	0.36	0.90	− 0.28	0.59	− 0.77	0.45	0.35
EOL-CDI item 14	0.88	0.14	1.07	1.43	1.09	1.19	0.33	0.39
EOL-CDI item 15	− 0.16	0.16	1.21	2.40	1.25	2.14	0.26	0.43
EOL-CDI item 16	0.32	0.15	0.84	− 2.70	0.79	− 2.63	0.56	0.42
EOL-CDI item 17	− 0.90	0.19	0.73	− 2.52	0.60	− 2.65	0.64	0.43
EOL-CDI item 18	0.98	0.14	0.97	− 0.73	0.92	− 0.94	0.42	0.39
EOL-CDI item 19	3.37	0.22	1.00	0.08	0.84	− 0.49	0.21	0.20
EOL-CDI item 20	1.20	0.14	0.90	− 2.21	0.93	− 0.82	0.45	0.37
EOL-CDI item 21	2.75	0.18	1.00	0.02	0.97	− 0.08	0.25	0.25
EOL-CDI item 22	− 0.31	0.16	0.72	− 3.50	0.64	− 3.27	0.66	0.43
EOL-CDI item 23	− 0.23	0.16	0.87	− 1.59	0.88	− 1.02	0.53	0.43
EOL-CDI item 24	− 0.13	0.16	0.91	− 1.09	0.85	− 1.35	0.50	0.43
EOL-CDI item 25	− 0.70	0.18	0.85	− 1.46	0.66	− 2.42	0.57	0.43
EOL-CDI item 26	0.82	0.14	0.97	− 0.63	0.98	− 0.23	0.42	0.39
EOL-CDI item 27	1.38	0.14	0.95	− 1.09	0.93	− 0.72	0.41	0.36
EOL-CDI item 28	1.98	0.15	0.90	− 1.64	0.80	− 1.57	0.41	0.32
EOL-CDI item 29	− 0.23	0.16	0.84	− 1.89	0.89	− 0.95	0.54	0.43
Mean ± SD			0.99 ± 0.15		0.97 ± 0.22			

*EOL-CDI* end-of-life care decision inventory, *MnSq* mean-square residual, *SE* standard error

MnSq of outside range > 1.3 or < 0.6, and Z-value outside range > 2.0 or <  − 2.0

**Table 5:** Standardized residuals’ variance in eigenvalue units of EOL-CDI final items (*N* = 238)

		Eigenvalue	Observed (%)	Expected (%)
EOL-CDI (21 items)	Total raw variance in observations	37.64	100.0	100.0
	Raw variance explained by measures	16.64	44.2	43.7
	Raw variance explained by persons	7.47	19.8	19.6
	Raw variance explained by items	9.17	24.4	24.1
	Raw unexplained variance (total)	21.00	55.8	56.3
	Unexplained variance in 1st contrast	2.04	5.4	9.7
	Unexplained variance in 2nd contrast	1.70	4.5	8.1
	Unexplained variance in 3rd contrast	1.56	4.1	7.4
	Unexplained variance in 4th contrast	1.53	4.1	7.3
	Unexplained variance in 5th contrast	1.38	3.7	6.6

*EOL-CDI* end-of-life care decision inventory

**Table 6:** Final version item fit statistics and point measure correlation of EOL-CDI (21 items) (*N* = 238)

EOL-CDI item	Logit	SE	Infit		Outfit		Point measure correlation	
			MnSq	Z	MnSq	Z	Observed	Expected
EOL-CDI item 1	− 3.49	0.41	0.91	− 0.18	1.20	0.50	0.36	0.35
EOL-CDI item 2	− 2.04	0.25	1.16	0.94	1.33	0.91	0.36	0.46
EOL-CDI item 3	− 2.74	0.31	0.87	− 0.55	1.38	0.80	0.43	0.40
EOL-CDI item 9	− 1.43	0.22	1.26	1.77	1.57	1.79	0.35	0.50
EOL-CDI item 10	− 0.57	0.18	1.34	2.87	1.45	2.28	0.33	0.52
EOL-CDI item 12	− 1.63	0.23	1.65	1.65	1.50	1.48	0.33	0.48
EOL-CDI item 14	0.96	0.15	1.28	4.34	1.48	3.81	0.29	0.48
EOL-CDI item 16	0.33	0.16	0.85	− 2.02	0.78	− 1.98	0.60	0.51
EOL-CDI item 17	− 1.04	0.20	0.70	− 2.62	0.58	− 2.09	0.67	0.51
EOL-CDI item 18	1.08	0.15	0.99	− 0.15	0.91	− 0.81	0.49	0.48
EOL-CDI item 19	3.63	0.22	1.01	0.14	0.96	0.02	0.24	0.25
EOL-CDI item 20	1.32	0.15	0.95	− 0.96	1.09	0.73	0.48	0.46
EOL-CDI item 21	2.98	0.18	1.04	0.42	1.25	0.89	0.26	0.31
EOL-CDI item 22	− 0.38	0.18	0.73	− 2.88	0.68	− 2.18	0.67	0.52
EOL-CDI item 23	− 0.29	0.17	0.89	− 1.19	0.98	− 0.07	0.58	0.52
EOL-CDI item 24	− 0.17	0.17	0.97	− 0.27	0.88	− 0.78	0.55	0.52
EOL-CDI item 25	− 0.81	0.19	0.84	− 1.38	0.66	− 1.85	0.62	0.52
EOL-CDI item 26	0.90	0.15	1.00	− 0.04	0.97	− 0.21	0.49	0.49
EOL-CDI item 27	1.52	0.15	1.01	0.73	1.04	0.35	0.42	0.44
EOL-CDI item 28	2.16	0.16	0.89	− 1.63	0.75	− 1.40	0.46	0.39
EOL-CDI item 29	− 0.29	0.17	0.85	− 1.61	0.88	− 0.74	0.60	0.52
Mean ± SD			0.99 ± 0.17		1.06 ± 0.30			
PCA of standardized residuals’ 1 factor eigen value = 2.04								

EOL-CDI, end-of-life care decision inventory; MnSq, mean-square residual; SE, standard error

MnSq of outside range > 1.3 or < 0.6, and Z-value outside range > 2.0 or <  − 2.0

**Table 7:** Known-groups validity of EOL-CDI (*N* = 238)*

Group		n	Mean ± SD	t	*p*
LST Act education	Yes	90	14.82 ± 3.31	4.57	< 0.001
	No	148	12.58 ± 4.19		
EOL care experience	Yes	159	13.83 ± 3.81	2.27	0.024
	No	78	12.58 ± 4.35		
LST Act practice	Yes	153	14.07 ± 3.58	3.12	0.002
	No	84	12.27 ± 4.54		

*Missing data excluded

*EOL-CDI* end-of-life care decision inventory, *EoL* end-of-life, *LST* life-sustaining treatment

Internal consistency was verified on 21 items through person separation reliability and person separation index (Table 8). As a result, the person separation index was 1.81, which was an acceptable level of 1.5 or higher, and the person separation reliability was 0.8, corresponding to Cronbach's alpha (KR-20) reliability of 0.8.

**Table 8:** Reliability of EOL-CDI (21 item)

	Separation reliability	Separation index
Item	0.99	8.17
Person	0.77	1.81
Cronbach’s alpha (KR-20)	0.81	

*EOL-CDI* end-of-life care decision inventory, *ACP* advance care planning

#### Final version

Through verification of the validity/reliability of the tool and discrimination/difficulty of items, it was finally confirmed as one dimension with 21 items. The item is responded to as 'Yes', 'No', or 'don't know'. The correct answer for each item is scored as 1 point, and other responses are scored as 0 points. The total score ranges from 0 to 21. Therefore, the higher the score, the higher the level of understanding about EOL care decisions. Other than total score, whether or not target people understand the contents of each item also meaningful. And, final version was named EOL Care Decision Inventory (EOL-CDI).

## Discussion

This study was conducted to present a tool that evaluates adults’ and clinical nurses’ understanding of EOL care decisions by updating and validating an existing tool. To achieve the study purpose, we carried out processes deemed necessary to secure readability, validity, reliability, and feasibility. All processes were focused on accordance with applicable/current law and actual clinical practice.

For the final version, the authors and expert panel members reviewed all the processes and unanimously agreed to keep the items of ‘definition of terminal and EOL state’ that was suggested to be excluded by Rasch analysis. The reason for retaining these two items was obvious, and that is, these are an indispensable prerequisite for understanding ACP, LST, hospice palliative care, and other issues related to EOL care decisions. Consequently, the final version has been named ‘inventory’ instead of ‘questionnaire’.

The two attributes of ‘terminal/EOL care’ and ‘ACP’ were included as one dimension in EOL-CDI. Although these two attributes can be considered as two different dimensions, one-dimensionality was supported by Rasch Model and it is encouraging result since they are equally important in the EOL care decision making process. Moreover, the LST act in Korea specifies these two contents as must-addressed contents in discussing EOL care.

Related to ‘terminal/EOL care’, 7 items of hospice palliative care, terminal/EOL period, and LST were included, and ‘ACP’ comprised 14 items of AD and POLST < See “Appendix 1” > . The content of EOL-CDI was validated through expert consultation and quantitative methods and the appropriate level of readability and difficulty/feasibility was ensured through CIs and difficulty/discrimination tests respectively. The overall reliability of the KR-20 was 0.8, which can be considered high [22, 23], so it is judged to be suitable to evaluate understanding level of different groups’. The 'unconscious state' was eliminated first in the process of revising 29 items in the 5 categories proposed in the draft into ‘Revised Version 2’. In fact, the unconscious state, similar to persistant vegetative state, was considered an omission from the draft version. However, some experts recommended keeping it because of its importance related to decision-making capacity. However, as a result of the analysis, it failed to show enough discriminating power, so it was eliminated. The definition of unconsciousness is "a state in which the ability to maintain an awareness of oneself and the environment is lost, while the responsiveness to environmental stimuli is significantly reduced" [24]. Unconsciousness is broadly used interchangeably with coma, which refers to a “severe unconscious state due to decreased cerebral activity” [25] and is often confused with persistent vegetative state, especially among the general population. As such, confusion increases further when it goes with complex neurological diagnostic requirements, so its exclusion from the tool is considered valid.

For the 'terminal/EOL care' related items, a total of 7 items of hospice palliative care, decision-making capacity, and LST were included. Each item was presented plainly at the basic level. As such, it can be seen that the statement of knowledge related to EOL care decisions does not need to be specific or detailed in light of the fact that this tool consists of items that briefly describe each topic. In particular, with regard to 'terminal/EOL', items of decision-making capacity remained, and it is meaningful considering that it is important legally and ethically in EOL care decisions. In fact, the EOL care decision path is completely different depending on the decision-making capacity. In addition, considering that decision-making capacity or consent capability is an important factor in making decisions about one's body [26], it is appropriate that these are included in the tool. In terms of ethics, the ability to make decisions about one’s own body is consistent with the bioethical principle of respect for humans and autonomy [27]. Decision-making capacity is particularly important in terminal/EOL care decisions, which is also a major condition of AD of the patient self-determination act (PSDA) in the United States [28]. In addition, the LST Act in Korea deals with decision-making capacity seriously, so it is essential to check its understanding level among all stakeholders.

In the case of LST, there are differences in the scope and contents according to scholars, groups, and countries. Among the diverse list of LSTs, we included two approaches (mechanical ventilation and cardiopulmonary resuscitation, CPR) on the notion of being unlikely to be changed or controversial. However, only CPR was included in the tool after analysing the Rasch model. Mechanical ventilation, CPR, and tube feeding are usually included in the scope of 'resuscitation' in an address of Pope Pius XII to anaesthesiologists [29], which is still an important reference for EOL care decisions. Nonetheless, tube feeding was excluded from the beginning because it was not specified in the LST Act in Korea. Compared to tube feeding, mechanical ventilation is listed in the Act and has become a major subject to decide on its application in EOL. For this reason, it is unexpected yet interesting that mechanical ventilation was also excluded from the draft after analysis. One possible answer can be found in an address of Pope Pius XII. In particular, Pope Pius XII distinguishes between ordinary means and extreme unction with regard to these medical treatments [29]. That is, presenting as an important criterion what serious burdens are entailed on patients or others according to people, places, times, and cultural contexts, overly strict obligations will be too burdensome for most humans, and pursuing higher and more important virtues can be difficult [29]. In other words, considering that the importance of the context in which the patient is placed is pointed out rather than the type of LST, it can be seen as disproving that the understanding of individual LST itself may not be important in decision-making.

Next, related to 'ACP', all 14 items related to AD and POLST presented in ‘Revised Version 2’ were maintained. AD and POLST are the documents prepared through ACP and are also specified in the LST Act. This is similar to the PSDA in the United States, but in PSDA, AD was the core content when it started. Since then, in the United States, POLST began to be activated in 2004 by forming a POLST advisory group due to low usage of AD [30]. Likewise, POLST was also introduced in Korea and became an important document in clinical settings as it was legislated. These two documents are relatively new to Koreans, and the terms are somewhat similar in Korean language, so they are likely to be confused, requiring a relatively detailed understanding of these documents. For that reason, items that required awareness, such as the definition, the subject and completion process, and clinical issues, were organized into the draft version. Subsequently, these topics were recognized as important in expert consultation, so it is natural that they are finally included in the tool. The final version of the EOL-CDI was confirmed via processes including meticulous reviews of literature and laws, clinical expert consultations, and qualitative and quantitative examinations. We attempted to present a feasible tool in a way that was scientifically and practically appropriate. And, we suggest that EOL-CDI be used as a checklist that can guide education related to EOL care decisions as well as a tool to evaluate the understanding level of diverse groups. When EOL-CDI is used for knowledge evaluation purposes, total score and item-specific answers are available for analysis.

The current study has a number of limitations. First, the survey respondents were clinical nurses, and additional researches are needed for further verification of the validity and reliability of the tool targeting diverse groups. Second, minimum clinical important difference was not available from this study and should be further estimated especially for intervention study. And finally, unidimensional IRT was performed in this study since study results revealed one dimensionality and two attributes at the same time. And this analytic approach might be debatable, considering multidimensional IRT analysis provides information on factor structure and the underlying pattern of item responses by combining the advantages of CTT and IRT [31]. When the entire project including adults and elderly population is completed in the near future, more concrete results would be possible.

## Conclusion

EOL care decisions are an important issue in Korea and can cause subtle, ethical and philosophical anguish in that the consequences are in contact with death. For that reason, self-determination has become more important, and, in order to practice self-determination, there are certain informations that people should be aware of. Therefore, we tried to present a tool to identify people’s understanding level about relevant informations by revising an existing tool developed before the law was enacted. The revised tool would help researchers and clinicians explore diverse population’s awareness level about issues related to EOL care decisions.

## Data Availability

The datasets generated and/or analysed during the current study are not publicly available due to restrictions applied to the availability of these data, which were used under licence for the current study. Data are, however, available from the corresponding author upon reasonable request.

## Appendix: EOL-CDI

**Table:**

HPC is care that helps terminally ill patients die naturally and comfortably	
Painkillers would be stopped in HPC situation	
Basic medical services, such as nutrition, are provided when receiving HPC	
A terminally ill patient does not have the ability to make decisions	
A dying patient does not have the ability to make decisions	
LST is the treatment to treat disease	
CPR is performed when the heart or breathing stops	
AD is available to adults aged 19 or older	
AD is a document that specifies whether or not an adult wants LST in case he/she loses his/her ability to make a decision in advance	
AD can be prepared by a family member on behalf of a patient	
A patient may designate an agent to make a medical decision on his/her behalf using AD	
AD must be prepared in the designated institution	
In order to prepare AD, you need professional help either from a doctor or a nurse	
AD can be changed or abolished at any time	
POLST is the document completed in advance about terminally ill or dying patients’ wishes for LST at EOL	
POLST should be completed after the doctor explains it directly to the patient	
POLST cannot be changed once it is completed	
POLST can be completed by the opinion of the family instead of the patient	
POLST is a document completed by a doctor	
Other documents (DNR) allow to be used instead of POLST	
After completion of POLST, all medical services including painkillers and antibiotics will be suspended	

*EOL-CDI* end-of-life care decision inventory, *EOL* end-of-life, *HPC* hospice and palliative care, *LST* life-sustaining treatment, *CPR* cardiopulomary resuscitation, *ACP* advance care planning, *AD* advance directives, *POLST* a physician’s order of life-sustaining treatment, *DNR* do-not-resuscitate

## Abbreviations

ACP: Advanced care planning
AD: Advanced directives
CI: Cognitive interview
CPR: Cardiopulmonary resuscitation
EOL: End-of-life
EOL-CDI: End-of-life care decision inventory
ER: Emergency room
HPC: Hospice palliative care
ICU: Intensive Care Unit
IRB: Institutional Review Board
IRT: Item response theory
ITC: Item-total correlation
KR-20: Kuder–Richardson formula 20
LST: Life-sustaining treatment
MnSq: Mean square residual
POLST: Physician’s Order of life-sustaining treatment
PSDA: Patient self-determination act
PVS: Persistent vegetative state
QOL: Quality of Life
SDM: Shared decision-making
